# Chimeric Virus-Like Particles and Capsomeres Induce Similar CD8^+^ T Cell Responses but Differ in Capacity to Induce CD4^+^ T Cell Responses and Antibody Responses

**DOI:** 10.3389/fimmu.2020.564627

**Published:** 2020-09-29

**Authors:** David J. Pattinson, Simon H. Apte, Nani Wibowo, Tania Rivera-Hernandez, Penny L. Groves, Anton P. J. Middelberg, Denise L. Doolan

**Affiliations:** ^1^Infectious Diseases Programme, QIMR Berghofer Medical Research Institute, Brisbane, QLD, Australia; ^2^Centre for Molecular Therapeutics, Australian Institute of Tropical Health & Medicine, James Cook University, Cairns, QLD, Australia; ^3^Australian Institute for Bioengineering and Nanotechnology, University of Queensland, Brisbane, QLD, Australia; ^4^School of Chemical Engineering, The University of Adelaide, Adelaide, SA, Australia

**Keywords:** malaria, vaccine, T cells, virus-like particle, capsomere, murine polyomavirus, chimeric, *Plasmodium yoelii*

## Abstract

Despite extensive research, the development of an effective malaria vaccine remains elusive. The induction of robust and sustained T cell and antibody response by vaccination is an urgent unmet need. Chimeric virus-like particles (VLPs) are a promising vaccine platform. VLPs are composed of multiple subunit capsomeres which can be rapidly produced in a cost-effective manner, but the ability of capsomeres to induce antigen-specific cellular immune responses has not been thoroughly investigated. Accordingly, we have compared chimeric VLPs and their sub-unit capsomeres for capacity to induce CD8^+^ and CD4^+^ T cell and antibody responses. We produced chimeric murine polyomavirus VLPs and capsomeres each incorporating defined CD8^+^ T cell, CD4^+^ T cell or B cell repeat epitopes derived from *Plasmodium yoelii* CSP. VLPs and capsomeres were evaluated using both homologous or heterologous DNA prime/boost immunization regimens for T cell and antibody immunogenicity. Chimeric VLP and capsomere vaccine platforms induced robust CD8^+^ T cell responses at similar levels which was enhanced by a heterologous DNA prime. The capsomere platform was, however, more efficient at inducing CD4^+^ T cell responses and less efficient at inducing antigen-specific antibody responses. Our data suggest that capsomeres, which have significant manufacturing advantages over VLPs, should be considered for diseases where a T cell response is the desired outcome.

## Introduction

The annual mortality rate of malaria is currently estimated at 405,000 people of whom 67% are children under 5 years of age (1). Eliminating the causative *Plasmodium* spp. parasite will likely require an effective vaccine (2) but conventional sub-unit vaccines strategies have thus far proved to be suboptimal. The identification and development of vaccine delivery platforms which induce long-lasting robust cellular and antibody immune responses is a global health priority. A specific goal is a vaccine against the pre-erythrocytic (sporozoite/liver) stage of *Plasmodium* sporozoites which would prevent both the clinical symptoms which develop during the blood stage, and the transmission of the diseases which occurs during the sexual stage.

The most advanced malaria vaccine candidate, RTS,S (also known as Mosquirix™), is a virus-like particle (VLP) comprising multiple copies of the *P. falciparum* circumsporozoite protein (*Pf* CSP) B cell repeats and some CD4^+^ and CD8^+^ T cell epitopes fused with recombinant hepatitis B surface antigen (RTS) and co-expressed with free hepatitis B surface antigen (S) (3), co-administered with AS01 adjuvant (4). However, although early clinical studies showed some protective efficacy in the first year after vaccination, it is now established that RTS,S induced protection is low and wanes quickly (5, 6). Significant research efforts have been directed at either improving the vaccine platform, or incorporating additional antigens to broaden the protective immune response. Additionally, ease and cost of manufacturing is an important consideration.

VLPs are formed when recombinant viral structural proteins assemble into a highly repetitive array which resembles the native form of the virus. These units contain no genomic material so are incapable of replication, but they are highly immunogenic and particularly good at inducing antibody responses without the need for additional adjuvants (7–9). VLP vaccines that have been licensed include hepatitis B virus and human papillomavirus where protection is mediated through neutralizing antibodies (10, 11). In those VLPs, the entire structural protein comes from the pathogen itself and their structure resembles the cognate native virus targeted by the vaccine. However, instead, a generic VLP independent of the pathogen target can be used to produce a chimeric VLP with antigenic epitopes inserted into regions of a flexible carrier virus, thereby making a vaccine platform which can theoretically target any organism [reviewed in (12–14)]. Chimeric VLPs have been shown to be effective at inducing robust antibody responses (12–14), but their efficacy in generating cellular responses has not been comprehensively investigated. *In vitro* studies have shown that chimeric hamster polyomavirus and SV40 VLPs incorporating T cell epitopes from mucin 1 and influenza, respectively, can induce activation of epitope-specific CD8^+^ T cells (15, 16). Further evidence of CD8^+^ T cell induction was reported using hamster polyomavirus in a mouse study which demonstrated *in vivo* clonal proliferation of transferred epitope-specific CD8^+^ T cells, tumor growth inhibition and protection from lymphocytic choriomeningitis virus (17). We recently reported that immunization of mice with murine polyomavirus incorporating defined T cell epitopes derived from the *P. yoelii* CSP antigen (*Py*CSP) induced robust CD8^+^ T cell responses as well as high antibody titres, but poor CD4^+^ T cell responses (18).

Murine polyomavirus VLPs are generated when VP1 structural proteins form pentameric capsomeres (19) which can then be chemically induced *in vitro* to self-assemble into a highly repetitive VLP containing 72 capsomeres (7, 9, 20). The crystal structure of the VP1 protein has been previously described (21) as well as the predicted structures of chimeric capsomeres and VLPs with the Group A Streptococcus J8 peptide epitopes (7). The formation of VLPs from the subunit capsomere components adds time and cost to vaccine production (22). Also, the introduction of foreign epitopes could interfere with the structural formation of the VLPs. These shortcomings could be overcome if the chimeric capsomeres themselves were sufficiently immunogenic so that the subsequent VLP production steps were not required. To address this, our colleagues constructed a truncated MuPy-VP1 which prevented the formation of VLPs, and further modified the protein with the addition of multiple epitope insertion sites to increase the resultant antigen to vector ratio (8). These truncated capsomeres were, however, less effective at inducing of antibody responses and required co-administration with adjuvants (7, 8, 23) including nanoparticles such as silica, poly (D,L-lactic-co-glycolic acid) (PLGA) and poly caprolactone (PCL) (23, 24). In another study, it was shown that antibody responses induced by capsomeres could be enhanced to levels similar to those of VLPs without adjuvants, but this required a 20–40 times increase in the capsomere dose (7, 25). However, IFN-γ cellular responses induced by capsomeres were similar to those of VLPs, even at low doses in a HPV16 L1 model (25). Additionally, both HPV16 L1 VLPs and their component capsomere induced robust cytotoxic CD8^+^ T cell responses which were capable of tumor regression in the absence of adjuvants (26).

Herein, we extended those studies to comprehensively compare chimeric murine polyomavirus VLP and capsomere constructs incorporating defined ${\text{CD}8}_{\left(280-288\right)}^{+}$ (27) and ${\text{CD}4}_{\left(59-79\right)}^{+}$ (28) T cell and B cell repeat epitopes (29) from *Py*CSP for capacity to induce robust CD8^+^ and CD4^+^ T cell responses and antibody responses. In addition, we evaluated the potential benefit of co-administration of poly(I:C) adjuvant with VLPs, and of including a heterologous DNA prime-boost regimen.

## Materials and Methods

### Oligonucleotides, Peptides and Plasmid DNA

For genomic insertion of epitopes into VLPs and capsomeres, complementary oligonucleotides for the *Py*CSP ${\text{CD}8}_{280-288}^{+}$ T cell (SYVPSAEQI), ${\text{CD}4}_{59-79}^{+}$ T cell (YNRNIVNRLLGDALNGKPEEK), and B cell repeat (QGPGAPQGPGAP) peptide epitopes were codon-optimized for *E. coli* expression then synthesized by GeneWorks (Adelaide, Australia).

For peptide stimulation in T cell assays- *Py*CSP CD8_280−288_ (SYVPSAEQI), CD4_59−79_ (YNRNIVNRLLGDALNGKPEEK) and B cell (QGPGAPQGPGAP) peptides were purchased from Mimotopes Pty Ltd (Victoria, Australia).

For plasmid DNA immunizations, plasmid DNA encoding full-length *Py*CSP (pVR2516) or empty vector without insert (pVR1020; Vical Inc, CA, USA) were commercially purchased from PureSyn Inc. (Malvern, PA, USA).

### Plasmid Construction

The plasmid pGEX-4T-1 (GE Healthcare Biosciences, UK) with the murine polyomavirus VP1 sequence (accession number M34958) was obtained from the Protein Expression Facility (PEF, University of Queensland). For the VLP platform, the VP1 sequence was modified by PEF by inserting an *Afe*I restriction enzyme site flanked with Glycine_4_-Serine linker sequences at position 293; this was designated pGEX-VP1-S4-G4S (7). For the capsomere platform, the VP1 sequence was truncated to remove the first 28 and last 63 amino acids, and restriction enzyme sites were inserted at positions 28, 85, 293, and 380; this construct was designated VP1ΔNΔC (8). Human codon-optimized *Py*CSP ${\text{CD}8}_{\left(280-288\right)}^{+}$ and ${\text{CD}4}_{\left(59-79\right)}^{+}$ T cell epitopes and the B cell repeat epitope sequences were individually inserted into the *Afe*I site in pGEX-VP1-S4-G4S and into positions 28, 293 and 380 of VP1ΔNΔC using standard molecular biology techniques and constructs were confirmed by Sanger sequencing.

### Expression and Purification of Chimeric Capsomeres and VLPs

Wild-type pGEX-VP1-S4-G4S and VP1ΔNΔC, or the chimeric constructs detailed above, were separately transformed into chemically competent *E. coli* Rosetta DE3 pLysS bacteria (Novagen, CA, USA). The GST-tagged VP1 proteins were expressed by bacteria culture in Terrific Broth and expression induced using 0.2 mM IPTG and purified as previously described (7, 20). Briefly, filtered supernatant from sonicated bacteria were purified using a 5 ml GSTrap HP affinity column (GE Healthcare, UK), then the GST tag cleaved using thrombin (GE Healthcare UK). Capsomeres were then isolated by size-exclusion chromatography using a Superdex 200 10/300 GL column (GE Healthcare, UK). Endotoxin levels were reduced to below 5 EU/ml using Vivapure Q maxi H ion exchange columns (Sartorius Stedim, Gottingen, Germany) (7). VP1 capsomeres were assembled into VLPs by dialysis against an assembly buffer (7, 20) and then against PBS (7); VP1ΔNΔC capsomeres were dialyzed only against PBS (7). The characterization of VLPs for this project has been previously reported (18). VLPs were analyzed using asymmetric flow field-flow fractionation coupled to multi-angle light scattering (AF4-MALS) and transmission electron microscopy to assess size distribution as previously described (30, 31).

### Immunization of Mice

Female BALB/c mice (*n* = 5/group) aged 6–7 weeks (Animal Resources Center, WA, Australia) were immunized three times at 3-week intervals by (i) subcutaneous injection (s.c.) of pooled VLPs or pooled capsomeres (pools comprising 10 μg of each CD8, CD4 and B cell chimeric construct) on the lower back near the base of the tail; (ii) intramuscular injection (i.m.) of plasmid DNA (100 μg) into the anterior tibialis; or (iii) s.c. injection of pooled peptides (pools comprising 30 μg of each CD8^+^, CD4^+^ or B cell peptide epitopes) on the lower back near the base of the tail. Capsomeres and peptides were co-administered with 50 μg of high molecular weight poly(I:C) adjuvant (Invivogen, USA); and VLPs were administered with or without this adjuvant, in parallel groups. Mice were immunized in both homologous and heterologous prime/boost regimens involving two priming doses of *Py*CSP plasmid DNA followed by a booster dose of capsomere, VLP or peptide. Negative control groups included PBS, wild-type capsomere with poly(I:C), or wild-type VLP with poly(I:C). The positive control group received three doses of *Py*CSP plasmid DNA. All murine experiments were approved by the QIMR Berghofer MRI Animal Ethics Committee and were conducted in accordance with the Australian Code of Practice for the Care and Use of Animals for Scientific Purposes (2004).

### Splenocyte Harvesting and *in vitro* Stimulation

Ten days after the final immunization, spleens were harvested and single cell suspensions generated by mechanical disruption and red blood cell lysis. For ELISpot, cytometric bead array (CBA) and intracellular cytokine staining (ICS) assays, 5 × 10^5^ splenocytes were then co-incubated with 1.5 x 10^5^ gamma irradiated (16,666 cGy) mouse B cell lymphoma A20 cells (ATCC TIB-208) which had been either DNA-transfected, peptide-stimulated, or untreated. Transfections with plasmid DNA encoding *Py*CSP (pVR2516) or empty vector (pVR1020) was achieved using the AMAXA Nucleofector system (Lonza, Switzerland) using Kit V and program C-25 with 5 × 10^6^ A20 cells per cuvette, following the manufacturer's protocol. Peptide stimulation with *Py*CSP CD8^+^_(280−288)_ and CD4^+^_(59−79)_ T cell epitopes, or these peptides combined with the B cell repeat epitope peptide. Cells were incubated in KD-MEM media comprised of Dulbecco's Modified Eagle's Medium (SAFC Global, USA) supplemented with folic acid (136 nM), L-asparagine (32 mM), L-arginine (67 mM), sodium bicarbonate (24 mM), HEPES (10 mM), β-2-mercaptoethanol (5 nM), L-glutamine (1.5 mM), penicillin (100 Units/L), streptomycin (100 mg/L), and 10% fetal calf serum.

### IFN-γ ELISpot Assay

IFN-γ ELISpot assays were conducted as previously described (18, 32). Briefly, MSIPS4510 multiscreen ELISpot plate (Merck Millipore, Germany) well were pre-coated with 10 μg/ml anti-mouse IFN-γ antibodies (BD Biosciences, USA), blocked with KD-MEM containing 10% FCS, and washed. Then splenocyte/A20 cultures in quadruplicate were incubated at 37°C and 5% CO_2_ for 40 h. Wells were washed and stained with 2 μg/ml biotinylated anti-mouse IFN-γ antibodies (BD Biosciences, USA), followed by 1 μg/ml streptavidin-HRP (BD Biosciences, USA). The assay was developed using AEC substrate (BD Biosciences, USA). Spots were counted using the AID ELISpot reader system (Autoimmun Diagnostika GmbH, Germany).

### Cytometric Bead Array

Splenocyte/A20 cultures were incubated at in 96-well U-bottom plates in 200 μl of complete media at 37°C and 5% CO_2_ for 72 h. Culture supernatant was collected and stored at −80°C prior to assay. Secreted IFN-γ, TNF, IL-1β, IL-2, IL-4, IL-5, IL-6, IL-10, IL-12p70, and IL-13 cytokines were analyzed using the mouse cytometric bead array flex kit (BD Biosciences, USA) following the manufacturer's protocol. Samples were acquired using a FACSArray instrument (BD Biosciences, USA) and data analyzed using the CBA array software (BD Biosciences, USA).

### Intracellular Cytokine Staining

Splenocyte/A20 cultures in 200 μl of complete media supplemented with 0.1% Golgi Plug (BD Biosciences, USA) in 96-well U-bottom plates were incubated for 6 h at 37°C and 5% CO_2_. Cells were stained with PE-Cy7 labeled anti-CD8^+^ (53-6.7) and BV510 labeled anti-CD4^+^ (RM4.5) antibodies before being fixed with 4% paraformaldehyde at room temperature (RT) for 15 min. Cells were then washed with permwash and stained with APC-labeled anti-IFN-γ (XMG1.2), PE-labeled anti-IL-2 (JES6-5H4) and FITC-labeled anti-TNF-α (MP6-XT22) antibodies diluted in Cytofix/Cytoperm (BD Biosciences, USA). All antibodies were purchased from Biolegend with the exception of anti-TNF-α which was purchased from eBioscience. Flow cytometric analysis was performed on a Fortessa 4 (BD Biosciences, USA). Post-acquisition data analysis was performed using FlowJo software version 10 (Treestar, USA).

### ELISA

Sera was collected from mice 14 days after immunizations 1 and 2, and 5 days after the final immunization. Nunc Maxisorp plates (Thermo Fisher Scientific, USA) were coated overnight with either *Py*CSP B cell repeat peptide linked to a polystyrene binding tag (33) with a glycine_4_ spacer (34) (Mimotopes, Australia) (5 μg/ml) or *Py*CSP recombinant protein (1 μg/ml) diluted in carbonate buffer. Wells were subsequently blocked with PBS containing 2% BSA. Triplicate wells of 2-fold serially diluted sera in PBS-BSA 0.1% were used for endpoint titrations, and sera diluted 1:400 for isotype screening. For IgG responses, wells were incubated with biotinylated donkey α-mouse IgG antibodies (Jackson ImmunoResearch Laboratories, USA) diluted 1:20,000 in PBS-BSA 0.1%, followed by incubation with streptavidin-HRP (BD Biosciences, USA) diluted 1:1,000 in PBS-BSA 0.1 and 0.2% Tween20. For IgG isotype responses, wells were incubated with HRP-conjugated goat anti-mouse IgG_1_, rabbit anti-mouse IgG_2a_, goat anti-mouse IgG_2b_, or goat anti-mouse IgG_3_ antibodies (Invitrogen, USA) all diluted 1:3,000 in PBS-BSA 0.1 and 0.2% Tween20. Wells were developed with tetramethylbenzidine (TMB) and stopped using TMB stop reagent (Sigma Aldrich, USA). Absorbance was measured at 450 nm using a VersaMax microplate reader (Molecular Devices, USA). Positivity was defined as OD_450_ value >3× standard deviations above the mean blank (no serum) values.

### Indirect Fluorescence Antibody Test (IFAT)

Sporozoite-specific antibodies were assayed by Indirect fluorescence antibody test (IFAT) using a protocol modified slightly from that previously described (35). Briefly, cryopreserved *P. yoelii* 17XNL sporozoites (Sanaria Inc., MD, USA) were centrifuged at 10,000 × *g* for 5 min then resuspended to 10^5^ sporozoites per ml of Medium 199 (Life Technologies, USA). Then, 10 μl was added to wells drawn on a microscope slide using a Barrier Pap pen and air dried at RT before long-term storage at −80°C. Prior to use, slides were thawed to RT in a desiccator cabinet. Pooled sera from each immunization group collected 5 days after the final immunization was diluted 1:400 in PBS with 2% BSA, then 10 μl was added to each well and incubated at 37°C in a humid box. Wells were gently washed with PBS then stained with 10 μl of FITC conjugated anti-mouse IgG antibodies (BD Biosciences, USA), diluted 1:30 in filtered PBS containing 0.005% Evans blue. Slides were incubated for 30 min in a humid chamber and then washed gently with PBS. A cover slide was mounted over PBS with 10% glycerol and slides viewed on an EVOS fluorescence microscope (Advanced Microscopy Group, USA) at x400 magnification.

### Statistical Analysis

Statistical analysis was performed using GraphPad Prism version 6.0 (GraphPad, CA, USA). Logarithmic transformed data of groups were compared by one-way analysis of variance (ANOVA) and Bonferroni's multiple comparison test. Statistical significance is reported as ^*^p < 0.05, ^**^p < 0.01, ^***^p < 0.001, and ^****^p < 0.0001.

## Results

### Capsomere and VLP Construction

The genomic insertion of *Py*CSP peptide epitopes into the murine polyomavirus VP1 protein for generation of VLPs (7) or within the truncated VP1 proteins for capsomeres (8) was confirmed by Sanger sequencing. Size exclusion chromatography fractions post-GST cleavage showed the formation of capsomeres as previously described (8), and these were subsequently used for capsomere immunizations. The VLP forming proteins were assembled *in vitro* and analyzed by AF4-MALS analysis, which showed the mean radius of the chimeric VLPs [wild-type, 21.00 ± 1.27 nm; CD8 VLPs, 20.51 ± 0.67 nm; CD4 VLPs, 21.07 ± 0.61 nm; B cell VLPs, 20.85 ± 0.67 nm (mean radius ± SD)] with minimal amounts of aggregation (18). Transmission electron microscopy showed similar morphology between all VLP groups (18).

### Capsomeres Induce Similar CD8^+^ T Cell IFN-γ Responses to VLPs

**Figure 1:**
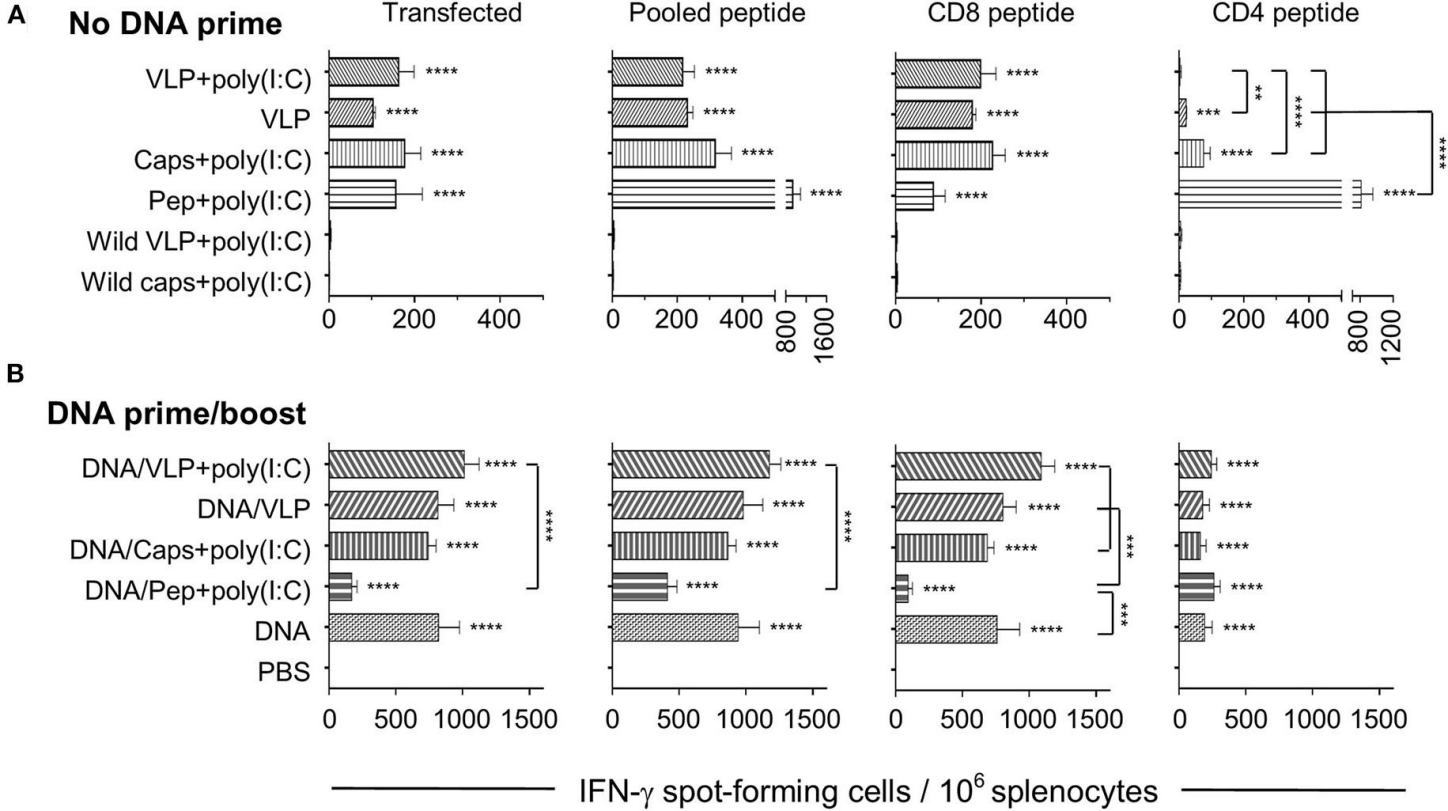
IFN-γ ELISpot responses induced by chimeric VLP or capsomere constructs. BALB/c mice (*n* = 5/group) received three immunizations with either **(A)** homologous s.c. injections of VLPs ± poly(I:C), capsomeres or peptides with poly (I:C), or **(B)** a heterologous DNA prime/boost regimen with two i.m. *Py*CSP plasmid DNA primes followed by a single s.c. boost with VLPs ± poly(I:C), capsomeres or peptides with poly (I:C). Seven days after the final immunization splenocytes were harvested and single-cell suspensions were stimulated *in vitro* for 40 h with irradiated A20 cells transfected with *Py*CSP plasmid DNA, or with irradiated A20 cells pulsed with either CD8 _(280−288)_, or CD4 _(58−79)_ peptides or a pool of both peptides plus the B cell repeat peptide. IFN-γ spot forming cells (SFCs) were quantified with data displayed as mean SFCs per 10^6^ splenocytes plus SEM. Statistical comparisons made to the PBS control group and between immunization groups with significance determined using one-way ANOVA followed by Bonferroni's post-test. **p < 0.01, ***p < 0.001, and ****p < 0.0001.

Significant antigen-specific IFN-γ responses were induced by homologous immunization with both capsomeres and VLP, when compared to their respective negative control, as detected after *in vitro* stimulation with *Py*CSP DNA transfected A20s or with pooled *Py*CSP peptides (Figure 1A). The amount of detected IFN-γ was not significantly different between capsomeres or VLP immunized groups (*p* > 0.05). The peptide immunizations also induced a robust IFN-γ responses which was dominated by the CD4 peptide stimulation.

Heterologous prime/boost immunization resulted in an ~5-fold increase in IFN-γ responses over the homologous regimens for both capsomeres and VLPs (Figure 1B). When compared to their homologous respective counterparts, the numbers of IFN-γ spot forming cells in the heterologous DNA prime/boost immunized mice were increased significantly for capsomeres (*p* < 0.001), VLPs and VLPs with poly(I:C) (*p* < 0.0001) when stimulated with the whole *Py*CSP antigen transfected into A20 cells, and capsomeres (*p* < 0.01), VLPs and VLPs with poly(I:C) (*p* < 0.0001) when stimulated *in vitro* with pooled peptides. This increase in IFN-γ responses was driven by the DNA prime as evidenced by comparison of the responses to those of DNA only immunized mice. Consistent with results from the homologous immunization regimens, IFN-γ responses induced by capsomeres and VLPs in the heterologous DNA prime/boost regimen were not significantly different.

**Figure 2:**
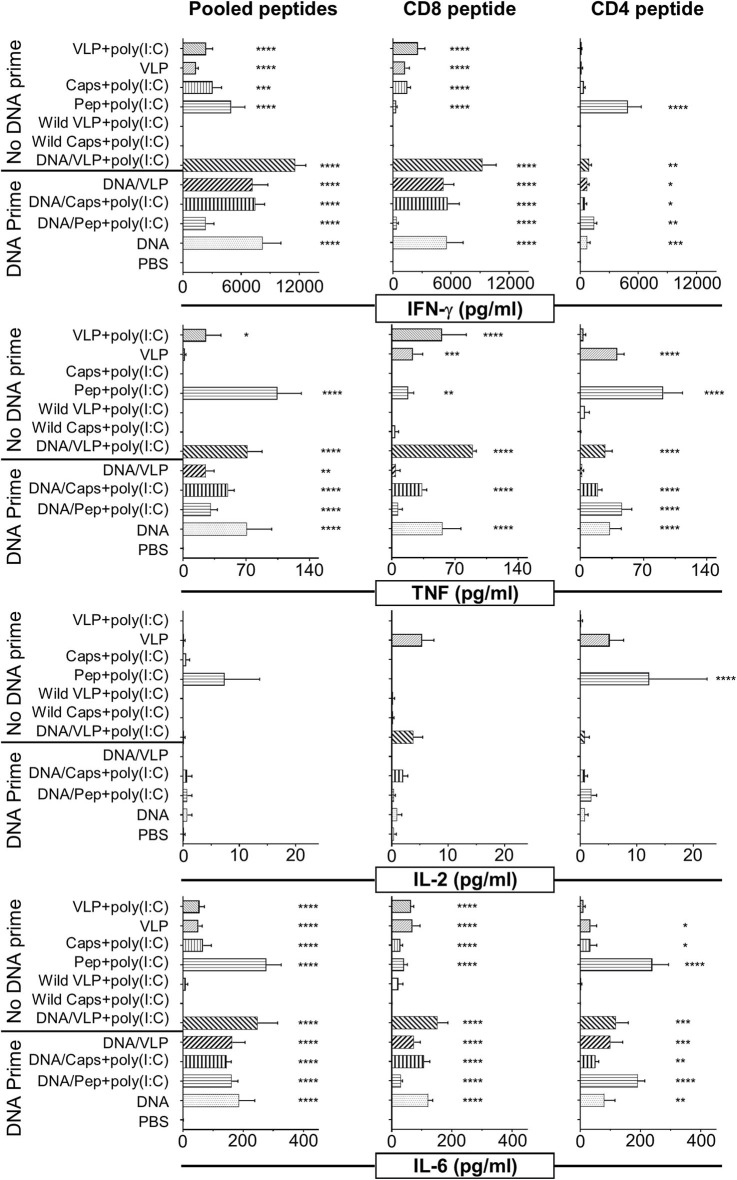
Cytometric bead array analysis of antigen-specific cytokine responses induced by immunization with chimeric VLPs or capsomeres. BALB/c mice (*n* = 5/group) were immunized with either a homologous (no DNA prime) or heterologous (DNA prime-boost) regimen as described in the legend to Figure 1. Seven days after the third immunization splenocytes were harvested and single cell suspensions stimulated with either the CD8^+^_(280−288)_, or CD4^+^_(58−79)_ T cell peptides or with pooled peptides including both peptides plus the B cell repeat peptide and incubated for 72 h at 37°C and 5% CO_2_. Culture supernatant was assayed using a cytometric bead array to quantify IFN-γ, TNF, IL-2, and IL-6 cytokine levels. Data are displayed as mean pg/ml + SEM for each cytokine with statistical comparisons made to the PBS control group with significance determined using one-way ANOVA followed by Bonferroni's post-test. *p < 0.05, **p < 0.01, ***p < 0.001, and ****p < 0.0001.

**Figure 3:**
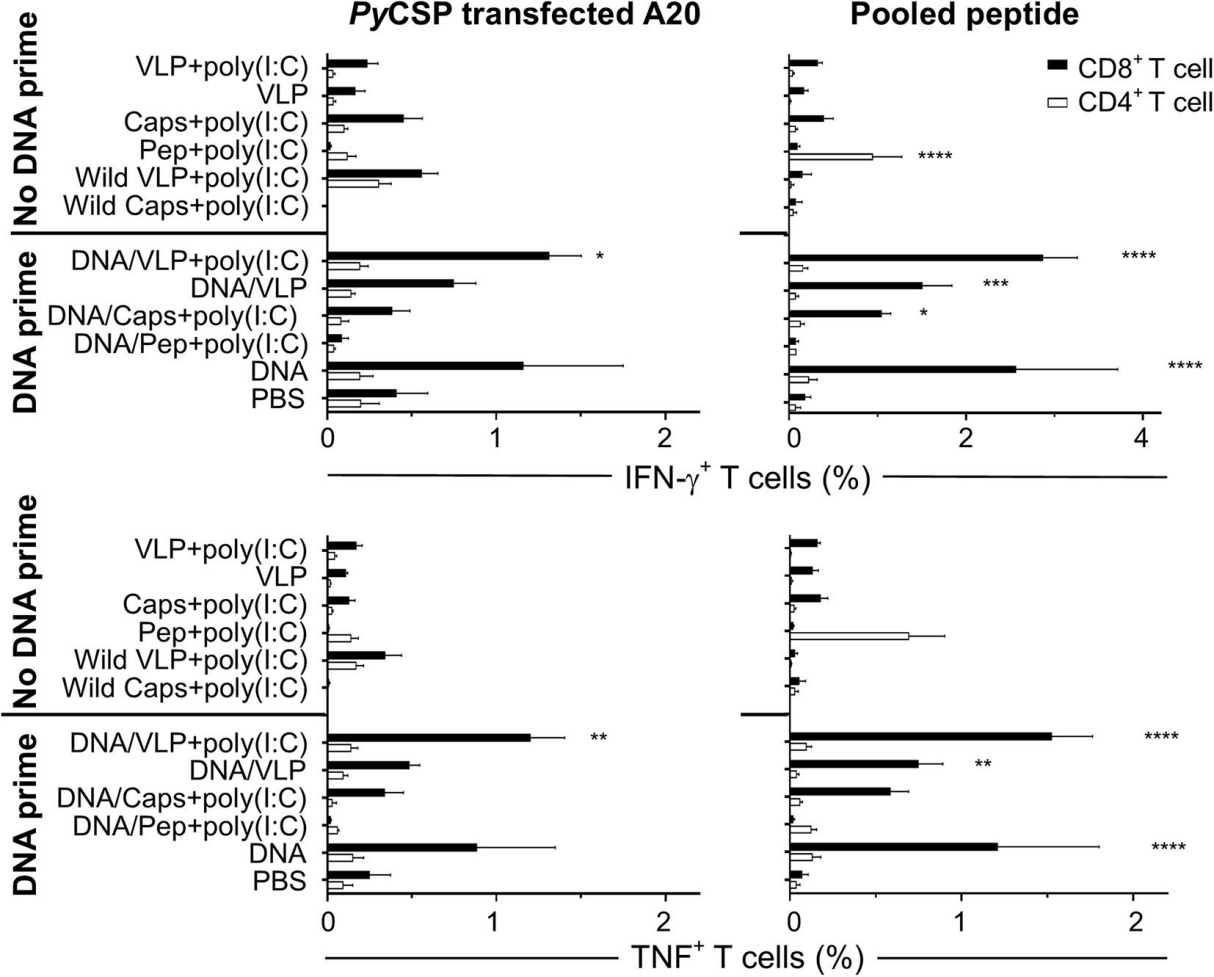
Flow cytometry frequency of stimulated splenocyte CD8^+^ and CD4^+^ T cells expressing IFN-γ and TNF post-immunization. BALB/c mice (*n* = 5/group) received three immunizations with either homologous (No DNA prime) or a heterologous DNA prime-boost regimen (DNA prime) as described in Figure 1. Seven days after the final immunization splenocytes were harvested and single-cell suspensions were stimulated *in vitro* by culturing them with irradiated A20 cells either transfected with *PyCSP* plasmid DNA or pulsed with *Py*CSP CD8^+^ and CD4^+^ T cell and B cell repeat peptides and incubated in for 1 h before adding Golgi Plug followed by a further 5 h incubation. Cells were stained for CD8 and CD4 receptors, then permeabilized and stained for IFN-γ and TNF cytokines then assessed by flow cytometry. The frequency of CD8 or CD4 positive cytokine-expressing cells are shown as the group mean + SEM (*n* = 5 mice/group) with CD8^+^ and CD4^+^ T cells represented by solid black bars or open bars, respectively. Statistical comparisons made to the PBS control group with significance determined using one-way ANOVA with Bonferroni's post-test. *p < 0.05, **p < 0.01, ***p < 0.001, and ****p < 0.0001.

To differentiate the responses observed with the pooled peptide stimulation *in vitro*, we also stimulated with either *Py*CSP CD8^+^ or CD4^+^ T cell peptides separately. That study showed that both the capsomere and VLP induced IFN-γ responses were predominantly associated with the CD8^+^ T cell peptide, with no significant difference between the capsomere and VLP immunized groups (Figures 1, 2). ICS was used to determine whether the CD8^+^ or CD4^+^ T cells were responsible for the production of IFN-γ (Figure 3). For both *Py*CSP capsomere and VLP immunized mice, IFN-γ was predominantly produced by CD8^+^ T cells. Similarly, in the heterologous prime/boost regimen, responses were directed against the CD8^+^ T cell epitope and apparently driven by the DNA component with responses similar to that induced by homologous DNA only immunization.

### Capsomeres Induce More Robust CD4^+^ T Cell IFN-γ Responses Than VLPs

Although capsomere and VLP platforms induced a very similar profile of CD8^+^ T cell responses, unexpectedly, they differed in ability to induce antigen-specific CD4^+^ T cell responses. Specifically, homologous capsomeres induced significantly higher IFN-γ responses than VLPs with poly(I:C) (*p* < 0.0001) when stimulated with the CD4^+^ T cell peptide (Figure 1A). This trend was also evident in the CBA analysis (Figure 2) and in the CD4^+^ T cell response detected by ICS (Figure 3), although the responses did not reach the level of significance. As observed for the CD8^+^ T cell response, the heterologous prime/boost was more effective than homologous immunization in inducing robust responses to the CD4^+^ T cell epitope (Figures 1B, 2). With this heterologous regimen, which included two priming doses of DNA, the vaccine-induced CD4^+^ T cell response for capsomeres and VLPs were comparable and did not differ significantly from that of three doses of *Py*CSP plasmid DNA alone. Nonetheless, the highest IFN-γ responses to the CD4^+^ T cell peptide were seen with homologous immunization with pooled peptides with poly(I:C) showing beneficial effects of the repeat peptide doses.

### Multiplexed Cytokine Responses

A broader spectrum of cytokine responses, including TNF, IL-2, and IL-6 as well as IFN-γ, were quantified in culture supernatant from *in vitro* immune assays using cytometric bead array (CBA) (Figure 2). The IFN-γ response profile was consistent with that detected by ELISpot (Figure 1). IFN-γ responses induced by capsomeres and VLPs were comparable and directed predominantly against the CD8^+^ T cell peptide, whereas the CD4^+^ T cell peptide stimulated cytokine profile observed by CBA was higher for capsomeres than for VLPs although these differences were not significant.

Capsomeres did not induce a TNF response for either the CD8^+^ or CD4^+^ peptide, but significant TNF responses were induced by VLP immunization and this was further increased by poly(I:C) adjuvant; these responses were preferentially directed against the CD8^+^ T cell peptide but were also significant for the CD4^+^ T cell peptide epitopes (Figure 2).

Significant IL-6 responses were induced by both capsomere and VLP groups with or without DNA priming, with a similar profile for both CD8^+^ and CD4^+^ T cell peptide epitopes.

In the peptide-immunized mice, responses for robust for IFN-γ, TNF, and IL-6 cytokines were preferentially directed to the CD4^+^ T cell peptide.

Negligible IL-2 levels were induced by any of the vaccine platforms tested (capsomeres, VLPs, or peptide) as assessed by ICS (data not shown) and CBA (Figure 2). The IL-1β, IL-4, IL-5, IL-10, IL-12p70, and IL-13 cytokine responses detected by CBA were also very low and no differences were identified between the two platforms (data not shown).

### Capsomeres Were Less Efficient Than VLPs at Inducing Antibody Responses

**Figure 4:**
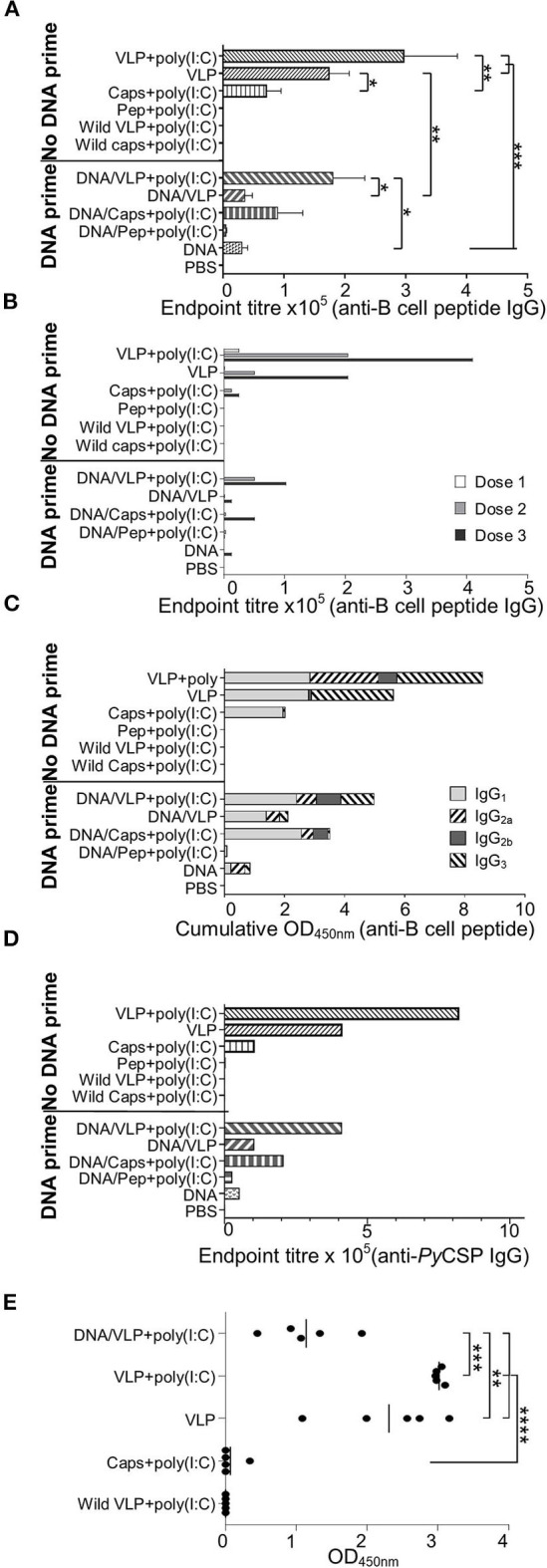
*Py*CSP-specific antibody responses induced immunization with chimeric VLPs or capsomeres. BALB/c mice (*n* = 5/group) were immunized with DNA, VLP, capsomeres or peptides in a three-dose homologous regimen or a heterologous DNA prime boost regimen as described in Figure 1. Sera collected 14 days after each immunization and 5 days after the final immunization were analyzed either individually or pooled, by ELISA using the *Py*CSP B cell repeat epitope linked to a polystyrene binding tag **(A–C,E)** or *Py*CSP protein **(D)** as capture antigen. **(A)** B cell epitope-specific endpoint total IgG antibody titres for individual mice with sera collected after the final immunization. Data is shown as mean ± SEM for each group. **(B)** B cell epitope-specific endpoint total IgG antibody titres for pooled sera collected following each immunization. **(C)** B cell epitope-specific IgG_1_, IgG_2a_, IgG_2b_, and IgG_3_ antibody isotype responses for pooled sera collected after the final immunization. Data is shown as a cumulative OD_450nm_ readout. **(D)** *Py*CSP antigen-specific endpoint total IgG antibody titer for pooled sera collected after the final immunization. **(E)** B cell epitope-specific IgG3 antibody responses for individual mice with sera after the final immunization. Data are for individual mice are shown as OD_450_ values with bars representing the group mean. Inter-group significance was determined using one-way ANOVA followed by Bonferroni's post-test. *p < 0.05, **p < 0.01, ***p < 0.001, and ****p < 0.0001.

ELISA assays against the PST-*Py*CSP B cell repeat peptide and *Py*CSP recombinant protein were performed using individual and pooled mouse sera collected 14 days after doses 1 and 2; and 5 days after final immunization (Figure 4). Consistent with previous studies, VLPs induced high anti-*Py*CSP B cell repeat IgG titres which were increased with the co-administration of poly(I:C) adjuvant (Figure 4A). Capsomeres induced moderate titres but, importantly, these were significantly less than VLPs with and without poly(I:C) (*p* < 0.01 and *p* < 0.05, respectively). There was a trend to increased antibody response with VLP administered with adjuvant compared to the non-adjuvanted VLPs but this was not statistically significant. However, the adjuvant effect was significant when VLPs were administered with a DNA prime (*p* < 0.05) indicating that with only a single VLP dose, the inclusion of adjuvant is beneficial and this benefit becomes less important after multiple doses (Figure 4B). A priming dose of DNA appeared to adversely affect the antibody induction, since responses in the heterologous prime-boost regimen tended to have lower titres than their homologous counterparts especially in the absence of poly(I:C) (*p* < 0.01).

We assessed the anti-*Py*CSP B cell peptide total IgG responses using pooled sera collected after each immunization to determine the effect of the number of immunizations within each regimen (Figure 4B). After each dose of VLPs, there was a trend to increased antibody titres when poly(I:C) adjuvant was co-administered.

Antibody isotypes contributing to the total IgG response reported above were also delineated, using pooled sera collected after the final immunization (Figure 4C). Immunizations with capsomeres, VLPs and VLPs with poly(I:C) each induced robust IgG_1_ responses which were similar for all groups, and the IgG2 response in the VLP-immunized mice was increased when administered with poly (I:C). VLP immunizations induced an IgG_3_ response which was absent in the capsomere-immunized mice. Compared to other immunization regimens, the homologous VLP immunization groups had higher levels of IgG_3_ but similar levels of IgG_1_ indicating that the second and third doses caused a shift in isotype responses toward an IgG_3_ bias. IgG_2a_ responses were increased with the inclusion of poly(I:C) with the VLP but this was not present in the capsomere with poly(I:C) immunized mice, indicating the difference was platform specific. Moderate IgG_2a_ responses were also present in the DNA group and the heterologous DNA prime groups.

To confirm that the vaccine-induced antibodies had affinity to the *Py*CSP protein, pooled sera collected after the final immunization was assayed using ELISA against recombinant protein (Figure 4D). The endpoint titres and profile were similar to that reported above for the B cell peptide (Figure 4A).

To confirm that the IgG_3_ response was consistent within groups, an ELISA against the *Py*CSP B cell repeat peptide was done using individual sera (1:400) collected after the final immunization (Figure 4E). Each VLP immunization group tested had significant amounts of IgG_3_ which was absent in chimeric capsomeres. The inclusion of poly(I:C) resulted in increased levels of IgG_3_ responses, and the homologous three-dose regimen significantly increased levels as compared to the heterologous prime/boost regimen (*p* < 0.001).

### Antibody Recognition of Native Antigens

**Figure 5:**
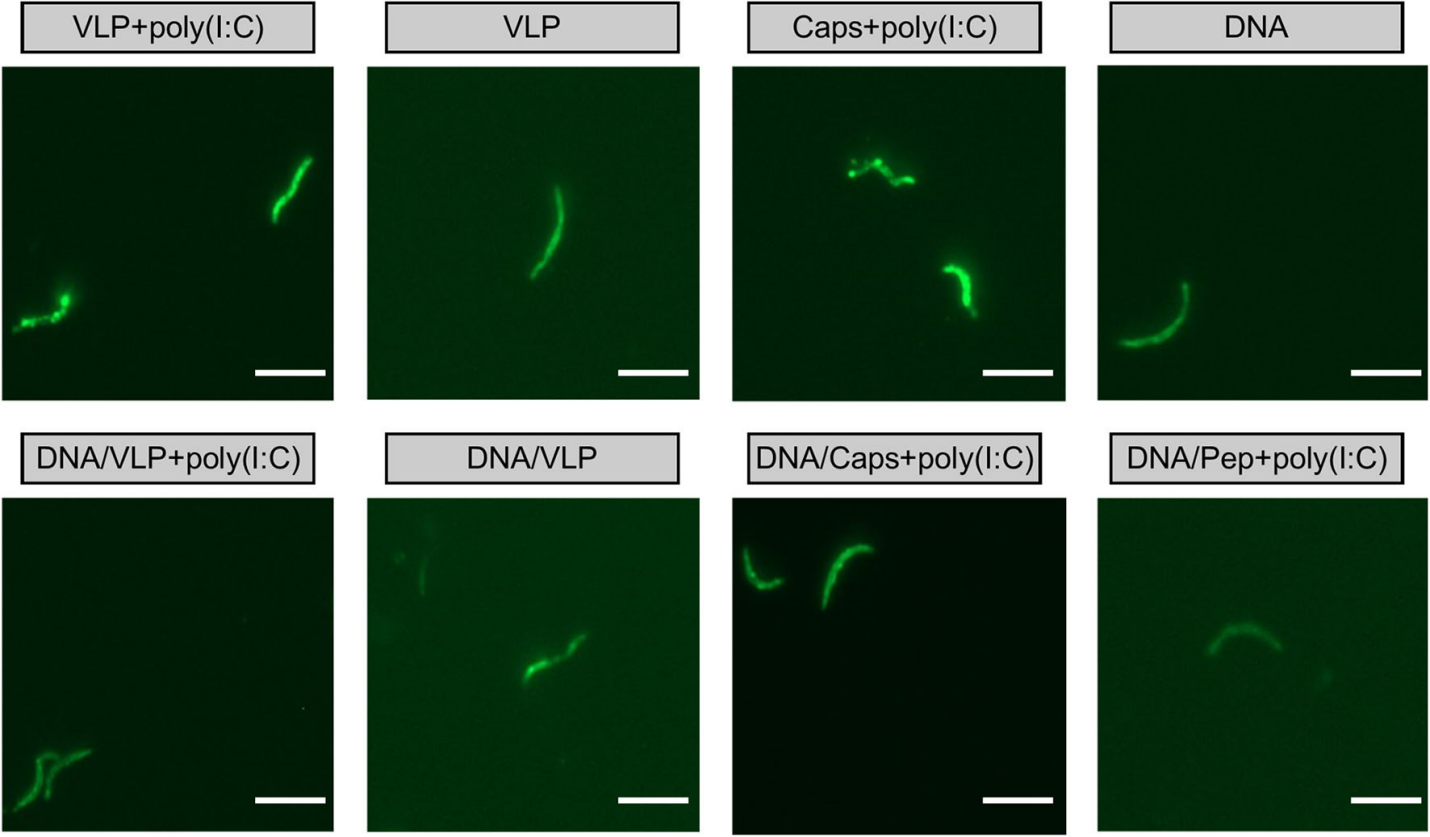
IFAT detection of anti-sporozoite antibodies induced by immunization with chimeric VLPs or capsomeres. Mice (*n* = 10/group) were immunized using a three-dose regimen with either homologous VLPs, capsomeres or *Py*CSP plasmid DNA; or a heterologous regimen with two *Py*CSP plasmid DNA doses followed by a single VLP or capsomere boost as described in Figure 1. Sera collected from mice 5 days after final immunization was pooled and assayed against *P. yoelii* 17XNL sporozoite-coated slides (1:400 dilution). Slides were stained using FITC-conjugated anti-mouse total IgG and viewed on an EVOS fluorescence microscope at x1000 magnification. Scale bar represents 10 μm.

Importantly, antibodies induced by immunized with capsomeres or VLPs with or without poly(I:C) adjuvant were able to recognize *P. yoelii* 17XNL sporozoites, as evidenced by surface staining in the homologous and heterologous immunization regimens (Figure 5).

## Discussion

The *Plasmodium* sp. CSP is the leading antigen target for sub-unit vaccines against malaria, and is the antigenic component of the virus-like particle RTS,S which is the most advanced human vaccine candidate against *Plasmodium falciparum* malaria. It was, therefore, a logical choice for evaluation of novel vaccine delivery platforms. We know that liver-stage protection from sporozoite challenge can be achieved by either CD8^+^ T cells (27, 36, 37), CD4^+^ T cell (38, 39) or antibody responses (29, 40). Thus, herein, we constructed chimeric VLPs and capsomeres incorporating a CD8^+^ T cell epitope, CD4^+^ T cell epitope, or B cell epitope derived from *Py*CSP in order to evaluate and compare the ability of capsomeres and VLP vaccine platforms to induce epitope-specific cellular and antibody responses.

Although the antibody-inducing capacity of chimeric murine polyomavirus VLPs and capsomeres has been well-established (7–9, 23), the ability of this vaccine platform to induce epitope-specific T cell responses has not previously been comprehensively evaluated. We set out to accomplish this. Our study established that capsomeres and VLPs induced similar and significant levels of antigen-specific IFN-γ responses and these responses were primarily directed against the immunodominant *Py*CSP CD8^+^ T cell epitope and produced by CD8^+^ T cells. This CD8^+^ T cell IFN-γ immune response is considered essential for protection (39, 41). Moreover, responses were increased when DNA priming was included as a heterologous regimen, as expected based on previous studies (18, 42–44). Interestingly, however, while both platforms were similar in capacity to induce an IFN-γ response, it was only the VLPs which evoked a TNF response, albeit at low levels, suggesting that VLPs may be better than capsomeres at inducing polyfunctional T cell responses.

In contrast to the comparability of the CD8^+^ T cell responses, VLPs and their subunit capsomeres differed significantly in capacity to induce CD4^+^ T cell responses and antibody responses. Unexpectedly, capsomeres proved to be the best platform at inducing responses to the CD4^+^ T cell epitope. The target *Py*CSP CD4^+^ T cell epitope _(59−79)_ was selected for study because of its the ability to induce functional CD4^+^ T cell populations of either T_H_1 or T_H_2 subsets associated with T cell proliferation responses or help for antibody responses (28). In our previous study, we could not detect any synergistic CD4^+^ T cell helper effect on antibody titres by co-administering a chimeric CD4 VLP (in a VLP pool) with B cell VLPs (18). In the current study, we were unable to show any CD4^+^ T cell helper responses as both capsomeres and VLPs were only administered in pools. We found that immunization with pooled peptides adjuvanted with poly(I:C) induced the most robust responses, however this is likely related to the dose of the target peptide epitope, as the positive control peptide pools incorporated 30 μg of the CD4 peptide whereas the amount of epitope presented in the chimeric capsomeres and VLPs was ~1.77 and 0.52 μg, respectively, per dose. This may also explain why the capsomeres were more effective than VLPs at inducing responses against this peptide epitope as one of the benefits of capsomeres over VLPs as a vaccine platform is the ability to present a higher antigen load, and in our study capsomeres achieved a 3-fold higher antigen dose than VLPs. It should be noted that the antigen dose difference was the same for the CD8^+^ T cell peptide epitope and we did not see similar increased immune responses against that antigen target. The ability of capsomeres to induce CD4^+^ T cell responses is an important advantage for vaccinology since vaccine platforms that preferentially induce CD8^+^ T cell responses rather than antibody responses are often poor inducers of CD4^+^ T cell responses (45). The essential role of CD4^+^ T cells for vaccine-induced protection against malaria has been demonstrated in CD4^+^ T cell-depleted sporozoite-immunized mice where an absence of CD4^+^ T cells resulted in reduced anti-sporozoite antibodies, a reduced effector capacity of CD8^+^ T cells, loss of protective efficacy (46).

It is well-established that VLPs are very effective at inducing antibody responses, perhaps due to their ability to cross-link B cell surface receptors (25) acting in a T cell independent manner (47, 48). Indeed, for all licensed VLP-based vaccines, protection is thought to be mediated through neutralizing antibodies (10, 11). Furthermore, studies using VLPs as well as their capsomere components with adjuvants showed them to be strong inducers of antibodies to inserted antigens (7–9, 23). Here, we have shown that VLPs with and without adjuvant were significantly better than capsomeres at inducing antigen-specific antibody responses, and that inclusion of poly(I:C) adjuvant increased antibody titres (49) with a skewed T_H_1 isotype profile. Importantly, these vaccine-induced antibodies were capable of recognizing the B cell repeat peptide, as well as recombinant *PyCSP* protein, and the parasite and IFAT showed antibody affinity to the surface of sporozoites establishing that they could recognize the whole parasite as well as the protein. The durability of the induced antibodies and their protective capacity has yet to be established. The value of anti-CSP antibodies has been shown by their ability to sterilely protect mice against sporozoite challenge (29), and by observations that anti-CSP IgG concentration and avidity contribute to RTS,S/AS01E-mediated protection (50) with anti-CSP antibodies estimated to prevent 32% of infections following RTS,S immunizations (51).

An interesting observation from our study is that the IgG_3_ antibody isotype observed with VLP immunizations contrasts with results obtained using the same platform incorporating the Group A Streptococcus J8 peptide, where IgG_1_ was the dominant IgG isotype and no IgG_3_ was detected (7). It appears, therefore, that the development of IgG_3_ is associated with the target epitope presented in the VLP structure rather than with the VLP platform itself. We had previously observed that an IgG_3_ response was induced by immunization with three doses of chimeric B cell epitope VLPs alone (our unpublished data), establishing that the CD8^+^ or CD4^+^ T cell chimeric VLPs were not responsible for the subclass, as seen in other platforms (52). It is curious that the IgG_3_ response was induced by immunization with VLPs but not capsomeres. IgG_3_ antibodies have previously been identified as important for protection against various pathogens (53, 54) including *Plasmodium* spp. parasites (35, 40), and IgG_3_ monoclonal antibodies raised against the *Py*CSP repeat (QGPGAP) protected BALB/c mice from a *P. yoelii* 17XNL sporozoite challenge (29).

There are many examples of enhanced immunogenicity and protection with DNA prime-boost regimens using various antigen delivery systems encoding the same antigen or epitope (44, 55–58) including VLPs (59) as a boost immunogen. Consistent with those reports, our data shows that the heterologous prime-boost regimen was better at inducing cellular responses than a homologous immunization regimen, for both capsomere and VLP platforms. The gain in cellular responses observed by including a plasmid DNA prime was, however, countered by a decrease in antibody titres. This is consistent with the known ability of plasmid DNA to preferentially prime a CD8^+^ T cell response and its poor ability to prime an antibody response (45).

Chimeric capsomeres are a promising vaccine platform which build on the established vaccine potential of VLPs, but are easier and cheaper to produce than VLPs (22), and can include more antigenic insertion sites because there is no reliance on structural VLP formation. Here, we show that capsomere and VLP platforms induced similar levels of CD8^+^ T cell responses but the capsomeres were significantly better at inducing epitope-specific CD4^+^ T cell responses than VLPs. This enhanced CD4^+^ T cell response may be of particular importance in the control of chronic viral infections (60), the maintenance of CD8^+^ T cells during prolonged viral infections (61) where CD4^+^ T cell help may be required for optimal CD8^+^ T cell activity (62) or for CD8^+^ T cell memory (63), or indeed for optimal responses against any pathogens where a CD4^+^ T cell vaccine induced response is required. On the other hand, since capsomeres have a limited capacity to induce antibody responses, VLPs would be the preferred platform for antibody mediated immunity. Given that most licensed vaccines target the induction of antibody response and the increasing interest at identifying vaccine platforms capable of inducing robust T cell responses, our data have important implications for the development of vaccines against those pathogens that have thus far proved challenging.

## Data Availability Statement

The datasets generated for this study can be obtained from the corresponding author upon reasonable request.

## Ethics Statement

The animal study was reviewed and approved by QIMR Berghofer Medical Research Institute Animal Ethics Committee.

## Author Contributions

DP, SA, DD, and AM contributed conception and design of the study. DP, NW, TR-H, and AM contributed to construction of virus-like particles and capsomeres. DP, SA, and PG conducted the mouse experiments. DP performed the statistical analysis. DP and DD wrote the manuscript. All authors contributed to manuscript revision, have read, and approved the submitted version.

## Conflict of Interest

The authors declare that the research was conducted in the absence of any commercial or financial relationships that could be construed as a potential conflict of interest.
